# Recognizing Genuine From Posed Facial Expressions: Exploring the Role of Dynamic Information and Face Familiarity

**DOI:** 10.3389/fpsyg.2020.01378

**Published:** 2020-07-03

**Authors:** Karen Lander, Natalie L. Butcher

**Affiliations:** ^1^Division of Neuroscience and Experimental Psychology, University of Manchester, Manchester, United Kingdom; ^2^School of Social Sciences, Humanities and Law, Teesside University, Middlesbrough, United Kingdom

**Keywords:** expression recognition, genuine and posed, dynamic information, face familiarity, face recognition

## Abstract

The accurate recognition of emotion is important for interpersonal interaction and when navigating our social world. However, not all facial displays reflect the emotional experience currently being felt by the expresser. Indeed, faces express both genuine and posed displays of emotion. In this article, we summarize the importance of motion for the recognition of face identity before critically outlining the role of dynamic information in determining facial expressions and distinguishing between genuine and posed expressions of emotion. We propose that both dynamic information and face familiarity may modulate our ability to determine whether an expression is genuine or not. Finally, we consider the shared role for dynamic information across different face recognition tasks and the wider impact of face familiarity on determining genuine from posed expressions during real-world interactions.

## Introduction

Face perception is a crucial part of social cognition, and on a daily basis, we encounter many faces. Faces convey characteristics of the viewed person, like their age, gender, emotional state, and identity. Face identity recognition is particularly important for social functioning as it enables us to identify a familiar person from an unknown individual. Previous research has revealed that factors including facial attractiveness, distinctiveness (Wiese et al., 2014), race (Meissner and Brigham, 2001), and facial motion (Lander et al., 1999) influence how well a face is recognized. Similarly, the ability to accurately determine another person’s emotional state is important for navigating day-to-day social interactions, for example, realizing whether a person is friendly or frightened, angry or sad. Previous research has shown that we use voice prosody (e.g., Wurm et al., 2001), body position (de Gelder, 2006), gait (Montepare et al., 1987), and facial expression (Adolphs, 1999) to determine emotional state.

Displayed facial expressions may reflect a genuinely felt emotion linked to an actual, remembered, or imagined event, for example, fear when scared or sad when remembering the death of a loved one. However, in some circumstances, facial expression may not reflect genuine emotion but instead be posed. Here, there may be no strong emotional experience, like smiling on cue or faking a surprised look. Alternatively, the expression displayed may mask the genuine emotion felt, like smiling when receiving a disappointing present. “Display rules” are rules learnt early in life that help determine the appropriate expression of emotion in different social contexts (Ekman and Friesen, 1969) and cultures (Matsumoto et al., 2009). Emotions may be amplified or de-amplified; they may be masked, neutralized, or simulated. Masking of emotions may be one way to recruit the help of others or otherwise gain a social advantage (Krumhuber and Manstead, 2009).

Research on facial expression processing has predominantly used static facial images taken at the expression “apex.” For example, Ekman and Friesen (1976) created a set of standardized static images of the “basic” facial expressions of happiness, sadness, fear, anger, disgust, surprise, and neutral. However, in the real world, facial expressions are dynamic in nature, rapidly changing over time. Interestingly, it is known that we are highly sensitive to dynamic information available from the face (Edwards, 1998; Dobs et al., 2014). Accordingly, sets of dynamic expressions have been developed (Amsterdam Dynamic Facial Expressions Set; ADFES; van der Schalk et al., 2011). It is important to consider the way in which expression sets are created. Typically, they are created by telling or showing the “actors” how to display prototypical expressions [based on facial action coding scheme (FACS) coding; Ekman and Friesen, 1978]. However, some research aims to capture genuine facial expressions that spontaneously occur as part of an emotional experience (see McLellan et al., 2010). Work on expression genuineness necessarily utilizes this method, with “genuine expressions” usually filmed in the lab. We return to consider the real-world application of such work, later in this article.

In this review, our overall aim is to explore the role of dynamic information in determining genuine from posed expressions. We start by outlining work investigating the recognition of face identity, highlighting the potential role for “characteristic motion signatures” (O’Toole et al., 2002). Next, we consider the role of dynamic information when recognizing facial expressions. Characteristic motion signatures may also be associated with emotional expressions and thus play a role in determining expression genuineness. Accordingly, we critically consider the difference between genuine and posed emotional expressions, in terms of the static- and dynamic-based cues available. Lastly, we consider the possible mediating effect of dynamic information and face familiarity when discriminating between genuine and posed expressions.

## Movement and the Recognition of Face Identity

Research has established that dynamic information is important when determining face identity (“motion advantage”; see Schiff et al., 1986; Knight and Johnston, 1997; Lander et al., 1999). Specifically, research has found that seeing a face move aids the learning of face identity (Pike et al., 1997; Knappmeyer et al., 2003; Lander and Bruce, 2003; Pilz et al., 2006; Lander and Davies, 2007; Butcher et al., 2011), identification of familiar faces (Knight and Johnston, 1997; Lander et al., 2001), and accurate and faster face matching (Thornton and Kourtzi, 2002). Dynamic facial information seems to be a particularly useful cue to identity recognition when viewing conditions are difficult, for example, when faces are presented in photographic negative (see Knight and Johnston, 1997; in a negative image, the pattern of brightness is reversed) or blurred (Lander et al., 2001). Also, dynamic information is useful when there is perceiver impairment, such as prosopagnosia (see Steede et al., 2007; Longmore and Tree, 2013; Xiao et al., 2014; Bennetts et al., 2015).

O’Toole et al. (2002) proposed several theoretical reasons why seeing a face move may facilitate identity recognition. These theories are not mutually exclusive and the extent to which they each account for the motion advantage may depend on whether the to-be-recognized face is unfamiliar or known. For unfamiliar faces, seeing a face move may help build robust face representations via structure-from-motion processes (“representation enhancement hypothesis”). However, for familiar faces, people may learn characteristic motion patterns associated with their identity, which act as an additional cue to identity (“supplemental information hypothesis”). Finally, social cues available from the moving face may attract attention to the identity-specific areas of the face, facilitating identity processing (“social signals hypothesis”). While both the representation enhancement and supplemental information hypotheses have received empirical support (e.g., Knappmeyer et al., 2003; Butcher et al., 2011), the plausibility of the social signals hypothesis is relatively unknown, as its predictions have received little attention. To summarize, dynamic information available from a moving face may be useful for both building new face representations and accessing established ones.

## Movement and the Recognition of Facial Expressions

While the motion advantage in identity recognition appears relatively robust, the effect of dynamic information on facial expression recognition is less consistent. Some research has shown that dynamic facial expressions are recognized more accurately (Cunningham and Wallraven, 2009; Trautmann et al., 2009) and rapidly (Calvo et al., 2016) than static facial expressions (see Krumhuber et al., 2013). However, other studies have found no difference between static and dynamic expression recognition (Kätsyri et al., 2008; Fiorentini and Viviani, 2011) or have only found a dynamic recognition advantage for some expressions (Fujimura and Suzuki, 2010; Recio et al., 2011).

One potential issue when comparing dynamic and static facial expression recognition is that static performance typically approaches ceiling, leaving little “room” to demonstrate any advantage. Interestingly, the usefulness of dynamic information for expression recognition is seen in studies that make recognition more difficult, through the use of point-light stimuli (Matsuzaki and Sato, 2008), subtle expressions (Ambadar et al., 2005), or by imposing time pressures (Zhongqing et al., 2014). Furthermore, Kamachi et al. (2001) found that changing the dynamic parameters of morphed expressions affected how well different expressions were recognized. As with identity recognition, dynamic facial information may support expression recognition in a flexible way, optimizing face perception when the task demands of everyday face-to-face interactions are such that static cues alone are not sufficient (Xiao et al., 2014).

In additional work supporting the distinction between recognition of moving and static expressions, Humphreys et al. (1993) report the case of an acquired prosopagnosic patient who could make expression judgments from moving (but not static) faces, consistent with the idea of at least partially dissociable static and dynamic expression processing. A number of neuroimaging studies have also investigated neural differences when viewing dynamic and static facial expressions (Kilts et al., 2003; Sato et al., 2004; Trautmann et al., 2009; Foley et al., 2012). Trautmann et al. (2009) found that dynamic faces enhanced emotion-specific brain activation patterns in the parahippocampal gyrus, including the amygdala, fusiform gyrus, superior temporal gyrus, inferior frontal gyrus, and occipital and orbitofrontal cortex. *Post hoc* ratings of the dynamic stimuli revealed better recognizability in comparison to the static stimuli (but see Trautmann-Lengsfeld et al., 2013). To summarize, much behavioral and neural work suggests that dynamic information can be useful in face expression recognition, particularly when recognition is difficult. However, this advantage is not unequivocally shown in the existing literature.

## Movement and the Recognition of Genuine From Posed Expressions

Increasingly, researchers have become interested in the distinction between genuine and posed facial expressions. Initially, research concentrated on static happy expressions (see Frank et al., 1993; Gunnery and Ruben, 2016). Here, genuine smiles (“Duchenne” smiles) are thought to involve crinkling around the eyes (“Crows feet”) caused by activation of the orbicularis oculi muscles. Posed smiles instead involve just an upturned mouth, created by contraction of the zygomatic major muscle. More recent work has investigated expression genuineness discrimination across a range of emotions.

Accordingly, McLellan et al. (2010) found that perceivers were able to distinguish between static genuine and posed happy, sad, and fear facial expressions. They also found that participants made valence judgments to words faster after viewing a genuine valence-congruent expression (i.e., smile before a positive word) compared to a posed expression. Additional support for differences between the perception of genuine and posed expressions comes from neuroimaging work which showed different patterns of neural activation (McLellan et al., 2012). However, findings by Dawel et al. (2015) suggest that the differences between genuine and posed expressions are less apparent than previously proposed. They found that both adults and children could discriminate genuine from posed happy expressions and adults were able to discriminate sad displays. However, neither group could discriminate between genuine and posed scared facial expressions. We conclude that most research, using static pictures, suggests that people can successfully discriminate between genuine and posed facial expressions in some circumstances – but that this ability may vary by expression and individual.

It is also important to consider the role of dynamic information in determining expression genuineness. Dynamic aspects of an expression may serve as useful cues when distinguishing genuine from posed expressions (Hess and Kleck, 1994; Gunnery and Ruben, 2016). Early research proposed that genuine smiles last between 500 and 4000 ms with posed smiles being either shorter or longer than this (Ekman, 2009). In addition, genuine smiles may have a slower onset speed and longer onset duration (Schmidt et al., 2006) than posed smiles. Recent research has begun to investigate the role of dynamic information in the recognition of expression genuineness across a range of facial expressions.

Interestingly, Namba et al. (2018) asked participants to judge whether viewed facial expressions were being depicted (posed) or experienced (genuine). Expressions (amusement, surprise, disgust, and fear) were shown as dynamic or static clips. For all expressions, genuine expressions were judged more as being experienced than posed. Importantly, participants were better at differentiating between genuine and posed expressions when dynamic than static. Similarly, Zloteanu et al. (2018) found that the use of moving stimuli improved the discrimination of surprise authenticity. We note that as with static images, overall performance on dynamic expression genuineness decisions may depend on the exact task used, what emotions are considered, the participants themselves, and so on. However, cues to expression authenticity may be present in the dynamics of the facial movement.

## Interdependence Between Face Familiarity and Face Movement in the Recognition of Expression Genuineness

We have already outlined research that suggests dynamic facial information is useful when determining the genuineness of facial expressions of emotion. Here, we further propose that there may be interdependence between face familiarity and face movement when determining expression genuineness.

In terms of face familiarity, it is known from neuroimaging studies that personal familiarity impacts on the response of neural systems involved in expression processing (Gobbini et al., 2004; Leibenluft et al., 2004). There is also some evidence that familiarity plays a role in the recognition of genuine emotional expressions, with performance seen to improve with familiarity (Wild-Wall et al., 2008; Huynh et al., 2010). However, other studies indicate a detrimental effect of familiarity on expression recognition in children (Herba et al., 2008) and some clinical populations (e.g., schizophrenia; Lahera et al., 2013). Thus, there is inconsistency regarding the role of familiarity on expression recognition.

Interestingly, research investigating the recognition of expression genuineness typically uses unfamiliar faces. This may be reflective of some real-life tasks, for example, in a criminal situation where the task is to determine whether an unfamiliar suspect is displaying a genuine expression or covering up a lie (Porter and ten Brinke, 2010). However, often, our interpretation of expression genuineness involves familiar people – for example, is our child genuinely happy or sarcastically smiling? Further research is needed to determine how face familiarity influences our ability to determine expression genuineness. We propose that for familiar faces, there may be additional cues that help us determine whether an expression is genuine or not, for example, a particular lop-sided smile associated with the genuine smile of a friend. Such idiosyncratic static-based cues may aid the distinction between genuine and posed smiles for this person. Thus, it is possible that face familiarity plays a mediating role in the recognition of genuine versus posed expressions, with better discrimination for familiar compared with unfamiliar faces.

It is also important to consider the possible interdependence between familiarity and dynamic information. When a face is familiar, characteristic motion patterns may act as an additional cue to identity. Indeed, the size of the motion advantage for face recognition is positively associated with face familiarity (Butcher and Lander, 2016). Such characteristic motion patterns may be linked to expressional movements. Thus, face familiarity may play a more prominent role when recognizing genuine from posed expressions using dynamic stimuli. For example, a friend may have a characteristic smile (present in the static image) but they may also have a characteristic way of smiling (dynamic characteristics). Here, cues to expression genuineness may be present in both the static- and dynamic-based parameters of a familiar person’s expression. To summarize, further work is needed to determine whether expression genuineness decisions are better for familiar than unfamiliar faces and whether this advantage is exaggerated for dynamic compared with static clips. In addition, we need to consider the interdependence between face familiarity, dynamic information, and expression genuineness.

## Concluding Comments and Future Directions

The literature reviewed demonstrates that dynamic information is useful for face identification (Lander et al., 1999), expression recognition (Krumhuber et al., 2013), and for expression genuineness judgments (Namba et al., 2018). Further, we propose a possible facilitative effect of face familiarity and face movement when determining expression genuineness. It is interesting to consider what other issues remain in this research area.

First, we propose a shared role for dynamic information across different face tasks. Much facial motion contains both identity-specific and expression information which, on an everyday basis, are processed simultaneously. Work is needed to determine whether neural models of face processing can account for the shared importance of dynamic information across different face processing tasks. According to Haxby’s neural account (Haxby et al., 2000; Haxby and Gobbini, 2011), there is one cortical pathway that processes invariant aspects of faces (identity and gender; Fusiform Face Area) and another that processes changeable aspects of faces (expression and eye gaze; posterior superior temporal sulcus face area; pSTS-FA). Pitcher et al. (2014) suggest that the dynamic motor and static components of a face are processed via dissociable cortical pathways. Alternatively, Bernstein et al. (2018) suggest an integrated neural model of face processing, with dorsal face areas (pSTS-FA) sensitive to dynamic and changeable facial aspects whereas ventral areas (Occipital Face Area and Fusiform Face Area) extract form information from both invariant and changeable facial aspects. Such neural accounts need to be integrated with behavioral work to better understand the shared role of dynamic information for the different face tasks we encounter in the real world.

Second, to fully understand the task of recognizing expression genuineness, it is necessary to know what information is required for this task. Low and high spatial frequencies play different roles in the perception of facial expressions (Vuilleumier et al., 2003). Low spatial frequencies carry global/configural information whereas high spatial frequencies convey localized/fine-grain information. Low and high spatial frequencies may also play different roles in the classification of expression genuineness (Laeng et al., 2010; Kihara and Takeda, 2019). Additional work is needed to isolate which spatial frequency aspects of faces are diagnostic of expression genuineness when shown as dynamic clips.

Finally, it is important to consider the collection and use of expressions used in recognition experiments. Genuine expressions using emotion elicitation methods in the lab may lack the spontaneity of genuine expressions in the real world (Smoski and Bachorowski, 2003). The selection of genuine expressions by the experimenter may also rely on the criteria used in posed expressions. We suggest that real world expressions may be more idiosyncratic and individualist than those collected in the lab, modulated by familiarity and context. Investigation of these issues is important so that we can further consider expression genuineness and the impact of familiarity and dynamic information.

## Author Contributions

All authors listed have made a substantial, direct and intellectual contribution to the work, and approved it for publication.

## Conflict of Interest

The authors declare that the research was conducted in the absence of any commercial or financial relationships that could be construed as a potential conflict of interest.
